# Impact of multiple treatment cycles with anti-CGRP monoclonal antibodies on migraine course: focus on discontinuation periods. Insights from the multicenter, prospective, *I-GRAINE* study

**DOI:** 10.1007/s00415-024-12192-9

**Published:** 2024-02-12

**Authors:** Piero Barbanti, Cinzia Aurilia, Gabriella Egeo, Stefania Proietti, Paola Torelli, Florindo d’Onofrio, Antonio Carnevale, Sofia Tavani, Bianca Orlando, Giulia Fiorentini, Bruno Colombo, Massimo Filippi, Stefano Bonassi, Sabina Cevoli, for the Italian Migraine Registry (I-GRAINE) study group

**Affiliations:** 1 Headache and Pain Unit, IRCCS San Raffaele, Via Della Pisana 235, 00163 Rome, Italy; 2 San Raffaele University, Rome, Italy; 3 Clinical and Molecular Epidemiology, IRCCS San Raffaele, Rome, Italy; 4 Unit of Neurology, Department of Medicine and Surgery, Headache Center, University of Parma, Parma, Italy; 5 Headache Center Neurology Unit, San Giuseppe Moscati Hospital, Avellino, Italy; 6 Headache Center San Filippo Neri Hospital, Rome, Italy; 7 Catholic University of Sacred Heart Rome, Fondazione Policlinico Universitario A. Gemelli, Rome, Italy; 8 Headache Unit, Department of Neurology, Scientific Institute San Raffaele Hospital, Vita-Salute University, Milan, Italy; 9 IRCCS Istituto delle Scienze Neurologiche di Bologna, Bologna, Italy

**Keywords:** Migraine, Treatment, Anti-CGRP mAbs, Discontinuation, Disease-modifier, Multiple treatments

## Abstract

**Objectives:**

While a single 12-month treatment cycle (TrC) with anti-CGRP mAbs is not disease-modifying for most patients, there is limited understanding of the effects of multiple TrCs on migraine course. We evaluated whether a second TrC might modify the migraine course by comparing the occurrence of migraine relapse after discontinuation of the second TrC to that following the cessation of the first TrC.

**Methods:**

In a real-life, multicenter, prospective study we considered all consecutive patients diagnosed with high-frequency episodic migraine (HFEM) or chronic migraine (CM) with > 3 treatment failures and treated with any anti-CGRP mAbs for ≥ 2 consecutive 12-month TrCs who were responders at week 12. The primary endpoint was the change in monthly migraine days (MMD) for HFEM or monthly headache days (MHD) for CM at the first month of treatment discontinuation after the second TrC (D2) compared to the first TrC (D1). Secondary endpoints included variations in monthly analgesic medications (MAM), Numeric Rating Scale (NRS), and Headache Impact Test (HIT-6) scores, ≥ 50%, ≥ 75%, and 100% response rates, and relapse from episodic migraine to CM and from no-medication overuse (MO) to MO at D2 vs. D1.

**Results:**

One-hundred-seventy-eight patients completed two 12-month TrCs with anti-CGRP mAbs. At D2, patients experienced a significant reduction in MMD (– 0.6, *p* = 0.028), MHD (– 2.6, *p* < 0.001), monthly analgesic medications (– 2.0, *p* < 0.001), and HIT-6 score (– 2.2, *p* < 0.001) compared to D1, indicating improved effectiveness. The ≥ 50% response rate at weeks 45–48 during the first TrC was 95.5%, while at weeks 45–48 of the second TrC was 99.4%. Corresponding rates at D1 was 20.2% whereas at D2 was 51.6% (*p* < 0.0001). No statistical difference emerged in ≥ 75% and 100% responders. The relapse rate from episodic migraine to CM at D2 was lower than at D1 (12.3% vs 30.4%; *p* = 0.0002) Fewer patients experienced relapse from no-MO to MO at D2 compared to D1 (29.5% vs 68.7%; *p* = 0.00001).

**Discussion:**

A second TrC with anti-CGRP mAbs demonstrated clinical improvements compared to the first one, as indicated by a milder migraine relapse at D2 compared to D1. Multiple TrCs with anti-CGRP mAbs could progressively modify migraine evolution by reducing CGRP-dependent neuroinflammatory nociceptive inputs to the brain.

## Introduction

Migraine is a chronic brain disorder characterized by paroxysmal headache attacks accompanied by vegetative symptoms [1]. Preventive treatment for migraine is recommended for patients experiencing at least 3–4 disabling migraine days per month [2]. This is crucial also to mitigate the risk of migraine chronicization and medication overuse (MO). The optimal duration for migraine treatment is debatable. Nevertheless, it is reasonable to assume that a prolonged treatment period is necessary to reverse the progressive functional and anatomical changes underlying migraine evolution [3]. Traditional migraine prevention, including beta-blockers, antiepileptics, calcium-channel antagonists, and tricyclics, has typically been limited to 4–6 months. This limitation stems from their overall low tolerability and high discontinuation rate [4]. The recent availability of drugs targeting the calcitonin-gene-related peptide (CGRP), such as anti-CGRP monoclonal antibodies (mAbs) and gepants, characterized by remarkable tolerability and safety, has allowed to extend migraine prophylaxis to 12–18 months [5].

Most patients discontinuing anti-CGRP mAbs within 12 months exhibit a progressive worsening of migraine over time [6–13]. This observation suggests that an effective modification of the migraine course could warrant a longer duration of preventive treatment and aligns with the approach taken in the therapy of other brain paroxysmal disorders, such as epilepsy and anxiety disorders [14, 15]. However, reimbursement issues imposed by local regulatory authorities limit an extended use of anti-CGRP mAbs in certain European countries. In Italy, for instance, their use must be discontinued in any patient after 12 months [16].

While a single 12-month treatment cycle (TrC) with anti-CGRP mAbs does not appear to be disease-modifying for most patients, there is limited understanding of the effects of multiple anti-CGRP mAbs TrCs on the course of migraine. Examining the evolution of migraine during discontinuation periods after at least two consecutive TrCs could offer a more comprehensive understanding of the actual impact of anti-CGRP mAbs on migraine progression.

To address this gap, we conducted a prospective, multicenter, cohort, real-life study to determine whether migraine relapse after discontinuation of the second TrC is less pronounced compared to that following the cessation of the first TrC.

## Methods

This is a multicenter, observational, prospective, real-life study started in December 2018 and is currently underway in 6 Italian headache centers. The study is a sub-project of the Italian Migraine Registry (I-GRAINE). The study received approval from the Institutional Review Board of the IRCCS San Raffaele Roma as coordinating center (RP 19/26), and subsequently the Ethics Committees of all participating centers approved the study. The study population included all consecutive patients diagnosed with HFEM or CM who had experienced documented failures with > 3 prior preventive migraine classes (according to the Italian Medicines Agency (AIFA) reimbursement criteria) and treated with erenumab, fremanezumab, or galcanezumab for ≥ 2 consecutive 12-month TrCs [16]. According to current AIFA regulation, in Italy, anti-CGRP treatment must be stopped for ≥ 1 month after a 12-month TrC.

**Fig. 1:**
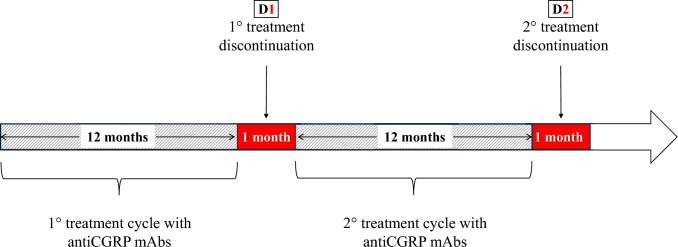
Flow chart of the study

As illustrated in Fig. 1, the first month of treatment discontinuation after the first TrC was defined as D1, while D2 represented the first month of treatment discontinuation after the second TrC. Following the acquisition of written informed consent, trained neurologists conducted face-to-face interviews using a web-based, standardized, semi-structured questionnaire to gather comprehensive sociodemographic and clinical data. Patients reported monthly migraine days (MMD)/monthly headache days (MHD), monthly analgesic medications (MAM), pain severity (using the Numerical Rating Scale NRS), migraine-related disability (using the Headache Impact Test HIT-6), and any adverse events in a paper–pencil diary over the study period.

The primary endpoint was the change in MMD for HFEM and MHD for CM at D2 compared to D1.

The secondary endpoints were:

- variations in MAM, NRS and HIT-6 scores at D2 compared to D1.
-  ≥ 50%, ≥ 75%, and 100% response rates at D2 compared to D1.
- Relapse rate from episodic migraine to CM and from no-MO to MO at D2 compared to D1.
- changes in MMD, MHD, MAM, NRS, and HIT-6 scores at:

- First TrC (weeks 45–48) compared to baseline.
- D1 compared to the first TrC (weeks 45-48).
- Second TrC (weeks 45–48) compared to the first TrC (weeks 45-48).
- Second TrC (weeks 45–48) compared to D1.
- D2 compared to the second TrC (weeks 45–48).

We excluded patients who had used onabotulinumtoxin A in the preceding three months, individuals with prior exposure to anti-CGRP monoclonal antibodies, and those with significant cardiovascular or cerebrovascular disorders. No additional preventive medications were initiated during the observation period. The study was not preregistered on any study registry site.

### Statistical methods

The characteristics of the study participants were summarized as frequencies and percentages for categorical variables, while mean and standard deviation (SD) were used for continuous variables. The Kolmogorov–Smirnov test was used to assess deviation from normality. Categorical variables were compared using the Chi-square test, with Fisher's exact test applied when the expected frequency was below 5. The comparison of continuous variables between HFEM and CM patients was done with the t-test for independent samples. All comparisons before–after treatment were performed with the t-test for paired samples. A *P* value < 0.05 was considered statistically significant. All statistical analyses were performed using IBM SPSS Statistics (Ver. 29.0).

## Results

One-hundred-seventy-eight patients completed two 12-month TrCs with anti-CGRP mAbs (erenumab: 133 pts; galcanezumab: 30 pts; fremanezumab: 15 pts). All patients used the same anti-CGRP mAbs during both the first and the second TrC, as therapeutic shifts are not permitted in Italy. AIFA reduced the initial discontinuation treatment duration from 3 to 1 month. In our patient cohort, the mean duration of D1 was 2.45 ± 0.9 months, with 129 patients undergoing a 3-month duration and 49 patients undergoing a 1-month duration. Conversely, the duration of D2 was consistently 1 month for all patients.

Most patients were females (73.6%) with a mean age of 48.6 years and affected by CM (77.5%). Patients with CM differed from those affected by HFEM for higher MAM, greater HIT-6 score, and more frequent use of concomitant medications (Table 1).

**Table 1:** Demographic and clinical features of migraine patients

	Number (%) or mean ± SD			*p*-values
	All patients	HFEM	CM	
Patients, *n*	178	40	138	
Age, years	48.5 ± 10.3	50.9 ± 9.6	47.8 ± 10.5	0.087
Females	131 (73.6)	31 (77.5)	100 (72.5)	0.665
BMI	23.1 ± 2.6	23.3 ± 2.6	23.1 ± 2.6	0.648
Age onset	16.9 ± 7.1	16.0 ± 5.9	17.2 ± 7.4	0.353
MMDs at baseline	11.8 ± 1.7	11.8 ± 1.7	–	–
MHDs at baseline	21.6 ± 5.2	–	21.6 ± 5.2	–
Monthly analgesic medications	19.6 ± 9.1	12.0 ± 2.1	21.8 ± 9.2	< 0.001
Medication overuse	112 (62.9)	–	112 (82.3)	–
Medication overuse duration, years	7.5 ± 6.1	–	7.5 ± 6.1	–
NRS score	7.9 ± 0.8	8.1 ± 0.6	7.8 ± 0.9	0.080
Unilateral pain	104 (61.2)	21 (60.0)	83 (61.5)	0.994
UAS	103 (59.2)	22 (57.9)	81 (59.6)	0.854
Ictal Allodynia	104 (60.8)	24 (64.9)	80 (59.7)	0.569
Dopaminergic symptoms	60 (35.5)	13 (36.1)	47 (35.3)	0.932
HIT-6 score	67.6 ± 5.7	65.3 ± 3.3	68.7 ± 6.0	0.003
Patients using concomitant migraine prophylaxis	71 (39.9)	12 (30.0)	59 (72.8)	0.147
Prior treatment failures, n				0.391
3–4	101 (60.5)	24 (66.7)	77 (58.8)	
> 4	66 (39.5)	12 (33.3)	54 (41.2)	
Pts with ≥ 1 comorbidity	74 (41.8)	19 (47.5)	55 (40.1)	0.517
Pts with psychiatric comorbidities	46 (26.0)	11 (27.5)	35 (25.5)	0.804
Pts using concomitant medications	170 (95.5)	34 (85.0)	136 (98.5)	0.008
Erenumab	133 (74.7)	26 (65.0)	107 (77.5)	
Galcanezumab	30 (16.9)	11 (27.5)	19 (13.8)	
Fremanezumab	15 (9.1)	3 (7.5)	12 (8.7)	

*HFEM* high frequency episodic migraine, *CM* chronic migraine, *BMI* Body Mass Index, *MHDs* monthly headache days, *MMDs* monthly migraine days, *NR*, Numeric Rating Scale, *UAS* Unilateral cranial autonomic symptoms, dopaminergic symptoms: presence during prodromes, headache stage or postdromes of have at least one of the following symptoms: yawning, somnolence, nausea, vomiting, mood changes, fatigue or diuresis, *HIT-6* Headache Impact Test-6

Primary endpoint (Table 2):

- *D2 vs. D1*: fremanezumab resulted in a significant decrease in both MMD (– 0.6, *p* < 0.028) and MHD (– 2.6, *p* < 0.001).

**Fig. 2:**
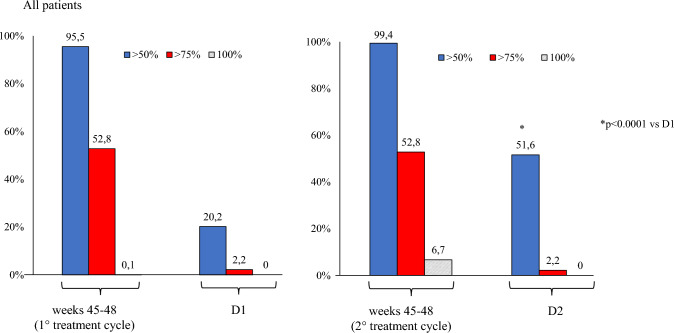
Proportion of patients with a ≥ 50%, ≥ 75% or 100% reduction in monthly migraine/headache days following treatment with anti-CGRP mAbs in all patients. D1: first month of treatment discontinuation after the first anti-CGRP treatment cycle; D2: first month of treatment discontinuation after the second anti-CGRP treatment cycle

**Fig. 3:**
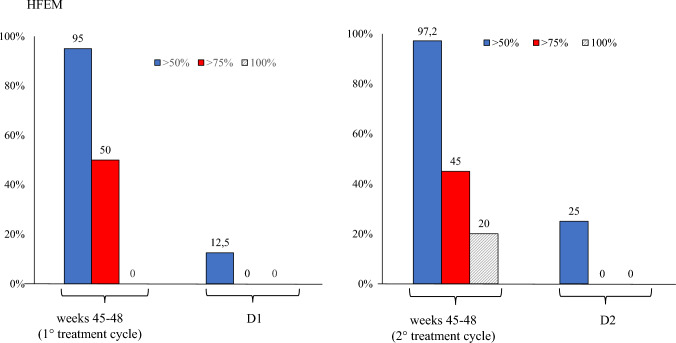
Proportion of patients with a ≥ 50%, ≥ 75% or 100% reduction in monthly migraine days following treatment with anti-CGRP mAbs in patients with high frequency episodic migraine (HFEM). D1: first month of treatment discontinuation after the first anti-CGRP treatment cycle; D2: first month of treatment discontinuation after the second anti-CGRP treatment cycle

**Fig. 4:**
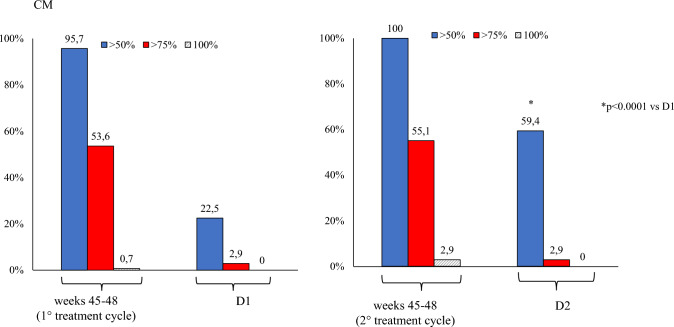
Proportion of patients with a ≥ 50%, ≥ 75% or 100% reduction in monthly headache days following treatment with anti-CGRP mAbs in patients with chronic migraine (CM). D1: first month of treatment discontinuation after the first anti-CGRP treatment cycle; D2: first month of treatment discontinuation after the second anti-CGRP treatment cycle

**Fig. 5:**
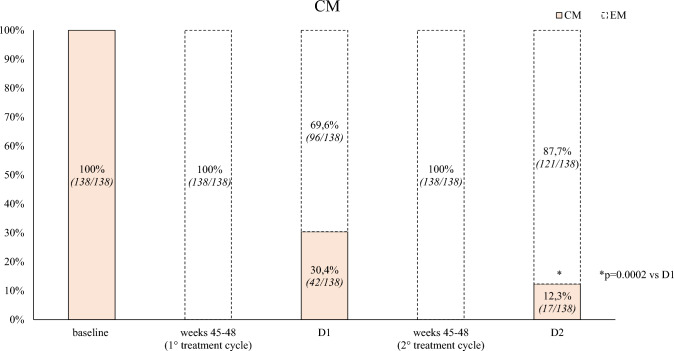
Proportion of patients remitting from chronic migraine (CM) to episodic migraine (EM) and vice-versa (colored bars: patients affected by chronic migraine, CM; white dotted bars: patients affected by episodic migraine, EM). D1: first month of treatment discontinuation after the first anti-CGRP treatment cycle; D2: first month of treatment discontinuation after the second anti-CGRP treatment cycle

**Fig. 6:**
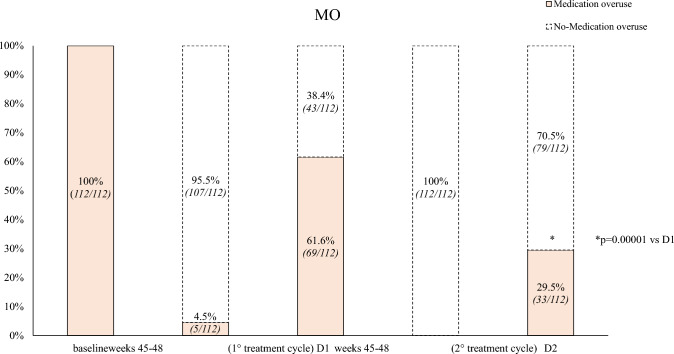
Proportion of patients remitting from medication overuse (MO) to no-MO and vice-versa (colored bars: patients with MO; white dotted bars: patients without MO). D1: first month of treatment discontinuation after the first anti-CGRP treatment cycle; D2: first month of treatment discontinuation after the second anti-CGRP treatment cycle

Secondary endpoints (Table 2):

- *D2 vs. D1*: patients showed a significant improvement in MAM use (– 2.0, *p* < 0.001), and HIT-6 score (– 2.2, *p* < 0.001). The NRS score demonstrated a reduction (– 0.2), though not statistically significant.
-  ≥ *50%,* ≥ *75%, and 100% response rates*: during the first TrC (weeks 45–48) were 95.5%, 52.8%, and 0.6%, respectively. During the second TrC (weeks 45–48) they were 99.4%, 52.8%, and 6.7%. The corresponding rates at D1 were 20.2%, 2.2%, and 0% whereas at D2 were 51.6%, 2.2%, and 0% (Figs. 2, 3, 4).
- *Relapse from episodic migraine to CM:* the relapse rate into CM at D2 (12.3%) was lower than at D1 (30.4%) (*p* = 0.0002) (Fig. 5).
- *Relapse from no-MO to MO:* the relapse into MO was less frequent at D2 (29.5%) compared to D1 (68.7%) (*p* = 0.00001) (Fig. 6) *and occurred only in previous overusers.*
- *First TrC (weeks 45–48) vs. baseline*: a significant (*p* < 0.001) reduction was observed in MMD (– 8.4), MHD (– 16.3), MAM (– 14.5), NRS (– 3.7) and HIT-6 (– 18.5) scores.
- *D1 vs*. *first TrC (weeks 45–48):* there was a significant increase (*p* < 0.001) in MMD (+ 5.6), MHD (+ 7.8), MAM (+ 6.6), NRS (+ 2.4), and HIT-6 (+ 11.1) scores.
- *Second TrC (weeks 45–48) vs. first TrC (weeks 45–48):* a significant reduction was noted in MHD (– 0.5, *p* = 0.003), MAM (– 1.1, *p* < 0.001), and HIT-6 score (– 1.6, *p* < 0.002).
- *Second TrC (weeks 45–48) vs. D1*: significant (*p* < 0.001) reductions were observed in MMD (– 5.9), MHD (-8.3), MAM (– 7.7), NRS (– 2.5), and HIT-6 (– 12.7) scores.
- *D2 vs. second TrC (weeks 45–48):* a significant increase (*p* < 0.001) was noted in MMD (+ 5.3), MHD (+ 5.6), MAM (+ 5.7), NRS (+ 2.3), and HIT-6 (+ 10.7) scores.

**Table 2:** Effects of the first and the second 12-month treatment cycle with anti-CGRP mAbs and of the first and the second 1-month discontinuation period on different migraine outcomes

Comparison	Parameter	Change (Mean ± SD)	*p*-value
First TrC (weeks 45–48) vs. baseline	MMD	– 8.4 ± 2.7	< 0.001
	MHD	– 16.3 ± 5.3	< 0.001
	Monthly analgesic medications	– 14.5 ± 7.7	< 0.001
	NRS	– 3.7 ± 1.3	< 0.001
	HIT-6	– 18.5 ± 9.7	< 0.001
	% CM	100% → 0%	< 0.001
	% MO	100% → 4.5%	
D1 vs. first TrC (weeks 45–48)	MMD	+ 5.6 ± 1.9	< 0.001
	MHD	+ 7.8 ± 3.1	< 0.001
	Monthly analgesic medications	+ 6.6 ± 4.3	< 0.001
	NRS	+ 2.4 ± 1.1	< 0.001
	HIT-6	+ 11.1 ± 6.3	< 0.001
	% CM	0% → 30.4%	< 0.001
	% MO	4.5% → 61.6%	< 0.001
Second TrC (weeks 45–48) vs first TrC (weeks 45–48)	MMD	– 0.3 ± 1.5	ns
	MHD	– 0.5 ± 2.0	0.003
	Monthly analgesic medications	– 1.1 ± 3.3	< 0.001
	NRS	– 0.07 ± 1.5	ns
	HIT-6	– 1.6 ± 6.6	0.002
	% CM	0% → 0%	ns
	% MO	4.5% → 0%	ns
Second TrC (weeks 45–48) vs. D1	MMD	– 5.9 ± 1.7	< 0.001
	MHD	– 8.3 ± 3.3	< 0.001
	Monthly analgesic medications	– 7.7 ± 3.6	< 0.001
	NRS	– 2.5 ± 1.6	< 0.001
	HIT-6	– 12.7 ± 6.5	< 0.001
	% CM	30.4% → 0%	< 0.001
	% MO	61.6% → 0%	< 0.001
D2 vs. second TrC (weeks 45–48)	MMD	+ 5.3 ± 1.7	< 0.001
	MHD	+ 5.6 ± 3.2	< 0.001
	Monthly analgesic medications	+ 5.7 ± 3.2	< 0.001
	NRS	+ 2.3 ± 1.9	< 0.001
	HIT-6	+ 10.7 ± 6.7	< 0.001
	% CM	0% → 12.3%	< 0.001
	% MO	0% → 29.5%	< 0.001
D2 vs. D1	**MMD**	**– 0.6 ± 1.7**	**0.028**
	**MHD**	**– 2.6 ± 3.6**	** < 0.001**
	Monthly analgesic medications	– 2.0 ± 3.6	< 0.001
	NRS	– 0.2 ± 1.4	ns
	HIT-6	– 2.2 ± 6.2	< 0.001
	% CM	0% → 12.3%	< 0.001
	% MO	4.5% → 29.5%	< 0.001

*TrC* 12-month treatment cycle with anti-CGRP mAbs, *D1* first month of treatment discontinuation after the first treatment cycle, *D2* first month of treatment discontinuation after the second treatment cycle, *MMD* Monthly Migraine Days, *MHD* Monthly Headache Days, *NRS* Numerical Rating Scale, *HIT-6* Headache Impact Test, *ns* not statistically significant

Primary endpoints are represented in bold

## Discussion

A single 12-month TrC with anti-CGRP mAbs does not appear to be disease-modifying, as it typically results in migraine relapse after treatment discontinuation [6–13]. However, this finding does not preclude the possibility that anti-CGRP mAbs could manifest disease-modifying effects when used over an extended period of time or after multiple TrCs. To achieve a more comprehensive understanding of the influence of anti-CGRP mAbs on migraine pathophysiological mechanisms, it is valuable to assess changes in migraine characteristics not only during consecutive TrCs but also to examine variations in migraine relapse during successive periods of TrC discontinuation. Indeed, discontinuation phases are particularly informative regarding the central effects of the treatment, as they coincide with a progressive reduction in the peripheral antinociceptive action of anti-CGRP mAbs [17]. In Italy, anti-CGRP treatment must be discontinued after 12 treatment months for at least 1 month, as mandated by AIFA reimbursement regulations [16]. Consequently, patients often undergo multiple TrCs, restarting anti-CGRP mAbs due to migraine recurrence. This provides an opportunity to evaluate the effects of multiple TrCs on migraine course, at least considering the first month following discontinuation.

The present prospective, multicenter, real-life study conducted in the Italian context suggests that a second 12-month TrC with anti-CGRP in patients with HFEM or CM adds substantial clinical benefits to migraine outcomes compared to the first one. Notably, the second TrC induced a more pronounced reduction in migraine frequency, analgesic use, and disability compared to the first TrC, particularly in patients with CM.

However, we underscore that the most crucial insights arise from comparing migraine parameters during the first and second treatment discontinuation periods. Migraine relapse at D2 was milder than at D1, as evidenced by a significantly lower increase in MMD/MHD, MAM, and migraine disability. Further, the proportion of ≥ 50% responders increased from 20.2% at D1 to 51.6% at D2 (HFEM from 12.5% to 25%; CM from 22.5 to 59.4%). Lastly, relapse from episodic migraine to CM and from no-MO to MO occurred less frequently during D2 compared to D1 (12.2 vs 30.4% and 29.5% vs 68.7%, respectively). These findings align with a gradual modification of the mechanisms underlying migraine evolution and progression across multiple anti-CGRP TrCs.

Almost all the patients presented a ≥ 50% response at 12 months during the first TrCs (95.5% and 99.4%, respectively). This very high proportion—in line with that reported by our group in a prospective 1-year study (91.3%)—is easily explained by the fact that our sample consists only of responders at 12 weeks, a requirement requested by AIFA to allow treatment continuation [18]. Notably, all responders during the first TrC were also responders during the second TrC.

The migraine course is typically fluctuating, leading over time to a complete or partial clinical remission in some patients, and to a persistent or progressive evolution in others [3]. The worsening of migraine represents the clinical manifestation of slowly evolving functional and anatomical changes in a susceptible brain within a predisposed individual, influenced by lifestyle, environmental factors, or comorbidities [3]*.* Repetitive peripheral sensitization induced by CGRP and other neuropeptides during migraine attacks leads to central sensitization with progressive adaptive changes including alteration in brain volume (thickening or gain), iron deposition, and white matter changes [3, 19]. Conversely, galcanezumab reverses cortical thickness in multiple brain areas among treatment responders, confirming the potential for a peripherally acting treatment to beneficially modify central migraine mechanisms [20].

Based on the aforementioned findings, it is evident that the potential to reverse the intricate pathophysiological mechanisms driving migraine progression lies primarily in prolonged migraine prevention strategies. The need for extending anti-CGRP mAbs treatment is also underscored by reports of late response (> 12 weeks) and ultra-late response (> 24 weeks) to anti-CGRP mAbs observed in a significant proportion (30%) of migraine patients [18, 21]. As astutely pointed out by Szabo et al., anti-CGRP mAbs exhibit a peripheral site of action in the meninges, providing quite rapid headache control, and a central mechanism of action, resulting in a slower migraine prophylactic effect. Therefore, prolonged (or multiple) TrCs with anti-CGRP mAbs are required to progressively act on the multiphasic mechanisms—operating at both cortical and subcortical levels—involved in migraine evolution [20].

The present study suggests that, instead of providing a definitive resolution, a second migraine TrC with anti-CGRP mAbs is likely to facilitate the recovery from maladaptive neural activity and counteract the intricate mechanisms leading to trigeminal and central sensitization. Clinically, this is demonstrated by a notable decrease in migraine frequency, analgesic intake, and disability, an increase in ≥ 50% responder rate, and a reduction in relapse to CM and MO during D2 compared to D1.

The use of data based on a single month of anti-CGRP mAbs treatment discontinuation (as required by Italian AIFA rules) represents the most relevant limitation of the present study. A deeper insight into the ability of multiple treatment cycles to modify the migraine course could be achieved by assessing the effects of treatment discontinuation for longer than 5 months, corresponding to the 5-half-life of these drugs. However, comparing the immediate consequences of anti-CGRP mAbs discontinuation over consecutive treatments may offer valuable insights into the slowly progressive biological effects exerted by the antagonism of CGRP-mediated peripheral and central sensitization provided by the treatment. Another limitation is that the study exclusively focused on patients with HFEM and CM, thereby excluding individuals with lower attack frequency. Additionally, most patients received treatment with erenumab. Lastly, since the average age of the patients was in their late forties—an age at which migraines might spontaneously improve—this could represent a confounding factor.

The strengths of the study include the substantial sample size, the prospective multicenter design, and the extensive series of clinical data collected through a shared web-based questionnaire within the framework of the Italian Migraine Registry.

In conclusion, our study suggests that a second TrC with anti-CGRP mAbs offers gradual clinical improvements compared to the first one, as indicated by a milder migraine relapse at D2 compared to D1, suggesting a potential to modify the course of migraine. While the logical approach to treating migraine involves prolonged prevention, the use of multiple TrCs with anti-CGRP mAbs could progressively modify migraine evolution by reducing CGRP-dependent neuroinflammatory nociceptive inputs impinging into the brain [20].

## Data Availability

The dataset used and analyzed during the current study is available from the corresponding author on reasonable request.
